# What Do We Have to Know about PD-L1 Expression in Prostate Cancer? A Systematic Literature Review. Part 5: Epigenetic Regulation of PD-L1

**DOI:** 10.3390/ijms222212314

**Published:** 2021-11-15

**Authors:** Andrea Palicelli, Stefania Croci, Alessandra Bisagni, Eleonora Zanetti, Dario De Biase, Beatrice Melli, Francesca Sanguedolce, Moira Ragazzi, Magda Zanelli, Alcides Chaux, Sofia Cañete-Portillo, Maria Paola Bonasoni, Alessandra Soriano, Stefano Ascani, Maurizio Zizzo, Carolina Castro Ruiz, Antonio De Leo, Guido Giordano, Matteo Landriscina, Giuseppe Carrieri, Luigi Cormio, Daniel M. Berney, Jatin Gandhi, Davide Nicoli, Enrico Farnetti, Giacomo Santandrea, Martina Bonacini

**Affiliations:** 1 Pathology Unit, Azienda USL-IRCCS di Reggio Emilia, 42123 Reggio Emilia, Italy; Alessandra.Bisagni@ausl.re.it (A.B.); Eleonora.Zanetti@ausl.re.it (E.Z.); Moira.Ragazzi@ausl.re.it (M.R.); Magda.Zanelli@ausl.re.it (M.Z.); mariapaola.bonasoni@ausl.re.it (M.P.B.); giacomo.santandrea@ausl.re.it (G.S.); 2 Clinical Immunology, Allergy and Advanced Biotechnologies Unit, Azienda USL-IRCCS di Reggio Emilia, 42123 Reggio Emilia, Italy; Stefania.Croci@ausl.re.it (S.C.); Martina.Bonacini@ausl.re.it (M.B.); 3 Department of Pharmacy and Biotechnology (FABIT), University of Bologna, 40126 Bologna, Italy; dario.debiase@unibo.it; 4 Fertility Center, Department of Obstetrics and Gynecology, Azienda USL-IRCCS di Reggio Emilia, 42123 Reggio Emilia, Italy; Beatrice.Melli@ausl.re.it; 5 Clinical and Experimental Medicine PhD Program, University of Modena and Reggio Emilia, 41121 Modena, Italy; Carolina.CastroRuiz@ausl.re.it; 6 Pathology Unit, Policlinico Riuniti, University of Foggia, 71122 Foggia, Italy; francesca.sanguedolce@unifg.it; 7 Department of Scientific Research, School of Postgraduate Studies, Norte University, Asunción 1614, Paraguay; alcideschaux@uninorte.edu.py; 8 Department of Pathology, University of Alabama at Birmingham, Birmingham, AL 35294, USA; scaneteportillo@uabmc.edu; 9 Department of Pathology, Case Western Reserve University, Cleveland, OH 44106, USA; alessandra.soriano@ausl.re.it; 10 Gastroenterology Division, Azienda USL-IRCCS di Reggio Emilia, 42123 Reggio Emilia, Italy; 11 Pathology Unit, Azienda Ospedaliera Santa Maria di Terni, University of Perugia, 05100 Terni, Italy; s.ascani@aospterni.it; 12 Haematopathology Unit, CREO, Azienda Ospedaliera di Perugia, University of Perugia, 06129 Perugia, Italy; 13 Surgical Oncology Unit, Azienda USL-IRCCS di Reggio Emilia, 42123 Reggio Emilia, Italy; Maurizio.Zizzo@ausl.re.it; 14 Molecular Diagnostic Unit, Azienda USL Bologna, Department of Experimental, Diagnostic and Specialty Medicine, University of Bologna, 40138 Bologna, Italy; antonio.deleo@unibo.it; 15 Medical Oncology Unit, Department of Medical and Surgical Sciences, University of Foggia, 71122 Foggia, Italy; guido.giordano@unifg.it (G.G.); matteo.landriscina@unifg.it (M.L.); 16 Department of Urology and Renal Transplantation, University of Foggia, 71122 Foggia, Italy; giuseppe.carrieri@unifg.it (G.C.); luigi.cormio@unifg.it (L.C.); 17 Barts Cancer Institute, Queen Mary University of London, London EC1M 5PZ, UK; daniel.berney@nhs.net; 18 Department of Pathology and Laboratory Medicine, University of Washington, Seattle, WA 98195, USA; jgandhi@uw.edu; 19 Molecular Biology Laboratory, Azienda USL-IRCCS di Reggio Emilia, 42123 Reggio Emilia, Italy; davide.nicoli@ausl.re.it (D.N.); enrico.farnetti@ausl.re.it (E.F.)

**Keywords:** PD-L1, prostate, cancer, epigenetic, DNA methylation, miRNA, immunotherapy, checkpoint inhibitors

## Abstract

Epigenetic alterations (including DNA methylation or miRNAs) influence oncogene/oncosuppressor gene expression without changing the DNA sequence. Prostate cancer (PC) displays a complex genetic and epigenetic regulation of cell-growth pathways and tumor progression. We performed a systematic literature review (following PRISMA guidelines) focused on the epigenetic regulation of PD-L1 expression in PC. In PC cell lines, CpG island methylation of the *CD274* promoter negatively regulated PD-L1 expression. Histone modifiers also influence the PD-L1 transcription rate: the deletion or silencing of the histone modifiers MLL3/MML1 can positively regulate PD-L1 expression. Epigenetic drugs (EDs) may be promising in reprogramming tumor cells, reversing epigenetic modifications, and cancer immune evasion. EDs promoting a chromatin-inactive transcriptional state (such as bromodomain or p300/CBP inhibitors) downregulated PD-L1, while EDs favoring a chromatin-active state (i.e., histone deacetylase inhibitors) increased PD-L1 expression. miRNAs can regulate PD-L1 at a post-transcriptional level. miR-195/miR-16 were negatively associated with PD-L1 expression and positively correlated to longer biochemical recurrence-free survival; they also enhanced the radiotherapy efficacy in PC cell lines. miR-197 and miR-200a-c positively correlated to PD-L1 mRNA levels and inversely correlated to the methylation of PD-L1 promoter in a large series. miR-570, miR-34a and miR-513 may also be involved in epigenetic regulation.

## 1. Introduction

As the discovery of novel biomarkers is urgently required to develop tailored therapies for various malignancies [1], increasing attention has been paid to immunotherapy targets such as Programmed death-1 (PD-1) and its ligand (PD-L1). They are type I transmembrane glycoproteins transcribed by *PDCD1* (located on chromosome 2) and *CD274* genes (located on chromosome 9), respectively [2,3]. PD-1 is expressed by activated T, B, NK cells, and monocytes, while PD-L1 is found on hematopoietic and non-hematopoietic cells: their expression is inducible by microenvironmental conditions [2,3]. Indeed, pembrolizumab monotherapy (anti-PD-1 monoclonal antibody) recently revealed good therapeutic activity, and the 2021 United States National Comprehensive Cancer Network (NCCN) guidelines have considered this drug indicated in selected prostate cancer (PC) patients [4,5]. So, at least in the US, patients with metastatic castration-resistant PCs showing microsatellite instability/mismatch-repair protein system deficiency (MSI-H/dMMR) could be treated with pembrolizumab as a second-line therapy setting or beyond. Unfortunately, the prevalence of MSI-H/dMMR PCs is low, and the administration of immunotherapy in PC patients is still limited in the current clinical practice [4,5].

Epigenetic alterations induce reversible and heritable changes, promoting differences in the expressions of oncogenes and oncosuppressor genes without changing the DNA sequence [6]. DNA methylation, covalent histone modifications, histone variants, microRNAs (miRNAs) effects, and chromatin-remodeling complexes are well-identified epigenetic mechanisms. However, PC displays a complex genetic and epigenetic regulation, leading to changes in cell growth pathways and overall tumor progression [6].

Epigenetic modifications accumulated by cancer cells influence gene expression and may contribute to tumor immune escape, also by targeting checkpoint inhibitors such as PD-L1. Moreover, epigenetic modulating drugs may be promising in reprogramming tumor cells, reversing epigenetic modifications, and cancer immune evasion [7,8,9,10,11].

Unfortunately, despite the increasing attention on molecular and epigenetic regulators in PCs, there is still limited evidence concerning the complex network of epigenetic factors modulating PD-L1 expression in PC [12]. In our systematic literature review, we have tried to describe the current knowledge on this topic.

## 2. Results

### 2.1. Literature Review Results

**Figure 1:**
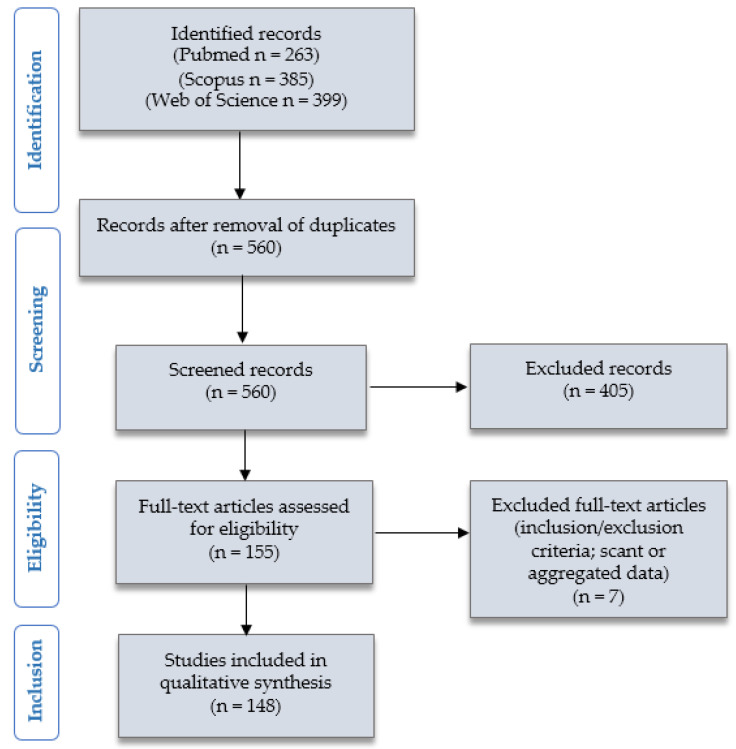
Review of the literature: PRISMA flow-chart.

Figure 1 presents the “Preferred Reporting Items for Systematic Reviews and Meta-Analyses” (PRISMA) (http://www.prisma-statement.org/, accessed on 8 May 2021) flow chart, summarizing the research method and results of our systematic literature review.

We identified 263 articles on PubMed (Available online: https://pubmed.ncbi.nlm.nih.gov, accessed on 8 May 2021), 385 articles on Scopus (Available online: https://www.scopus.com/home.uri, accessed on 8 May 2021), and 399 articles on Web of Science databases (Available online: https://login.webofknowledge.com, accessed on 8 May 2021). After duplicates exclusion, 560 records underwent a screening of titles and abstracts. 155 articles were considered eligible, as they seemed to report clinic-pathologic studies on human patients or experimental research on pre-clinical models (tumor cell lines, mouse models, etc.) investigating the role of PD-L1 in PC. After reading the full texts of all these papers, 7 articles were excluded for being unfit according to the inclusion criteria or for presenting scant or aggregated data. 148 articles were finally included in our study [5,12,13,14,15,16,17,18,19,20,21,22,23,24,25,26,27,28,29,30,31,32,33,34,35,36,37,38,39,40,41,42,43,44,45,46,47,48,49,50,51,52,53,54,55,56,57,58,59,60,61,62,63,64,65,66,67,68,69,70,71,72,73,74,75,76,77,78,79,80,81,82,83,84,85,86,87,88,89,90,91,92,93,94,95,96,97,98,99,100,101,102,103,104,105,106,107,108,109,110,111,112,113,114,115,116,117,118,119,120,121,122,123,124,125,126,127,128,129,130,131,132,133,134,135,136,137,138,139,140,141,142,143,144,145,146,147,148,149,150,151,152,153,154,155,156,157,158,159]. Further details are available in Section 4.

### 2.2. Epigenetic Regulation of PD-L1 Expression: Pre-Clinical Models

PD-L1 expression is under epigenetic control, as demonstrated by functional and correlation studies on PC cell lines (Table 1 and Table 2) [7,14,26,60,73,115,116,123,127,159]. 

In PC cell lines (Table 1), the methylation of CpG sequences in the *CD274* (PD-L1) gene promoter by DNA methyltransferases (DNMTs) negatively regulated PD-L1 expression [7]. To inhibit PD-L1, Li et al. [7] used recombinant constructs expressing the C-terminal domains of DNMT3a and/or DNMT1 fused with a zinc finger domain specifically binding to the PD-L1 promoter (Ad-ZF-DNMT3aC-1C, Ad-ZF-DNMT3aC, Ad-ZF-DNMT1C). Human PC cell lines (DU145) treated with Ad-ZF-DNMT3aC-1C showed a significant reduction in PD-L1 expression when compared to Ad-ZF-DNMT3aC or Ad-ZF-DNMT1C alone [7]. 

Histone modifiers also influence the PD-L1 transcription rate in PC cell lines: data suggested that the deletion or silencing of the histone modifiers *MLL3* and *MML1* may positively regulate PD-L1 expression [14,60]. Xiong et al. [60] found that MLL3 bound to the PD-L1 enhancer, while MLL3 depletion decreased the binding of the methylated histone H3 on Lysine 4 in the PD-L1 enhancer and Pol II Ser5p in the PD-L1 promoter. Moreover, it impaired mouse xenograft growth, decreasing the response to the PD-L1 antibody treatment.

The transcription factor IRF-1 could recruit p300 to the *CD274* promoter, inducing the acetylation of histone H3 and increasing *CD274* transcription [116]. In PC cell lines, protein levels of the histone deacetylase HDAC1 negatively correlated to PD-L1 levels, while there was no correlation between HDAC2/3 and PD-L1 [116].

Functional studies (Table 2) revealed that treatments with epigenetic drugs promoting a chromatin-inactive transcriptional state (such as bromodomain or p300/CBP inhibitors) [116,123,127] induced a reduction of PD-L1 expression; conversely, epigenetic drugs inducing a chromatin-active state (i.e., HDAC inhibitors) increased PD-L1 expression [116]. In an experimental study [116], p300 inhibitors (but not anti-PD-L1 antibodies) significantly enhanced the efficacy of HDAC inhibitors on limiting tumor progression by blocking the HDAC inhibition-induced PD-L1 expression.

Class I and II HDAC inhibitors such as SAHA (vorinostat) and LBH589 (panobinostat), as well as IFN-γ, significantly increased *CD274* expression in PC cell lines. RNA polymerase II was also enriched at the *CD274* promoter after HDAC treatment [116]. A485 may enhance the efficacy of treatments with anti-PD-L1 antibodies, decreasing the PD-L1 expression and reducing the exosomal PD-L1 secreted by PC cell lines; the combined administration of these drugs inhibited the androgen-independent metastatic tumor growth in syngeneic PC models [116].

The histone methylation regulator *WDR5* (WD repeat-containing protein 5) was overexpressed in PCs with advanced clinic-pathological features and seemed important for PD-L1 transcription [14]. In PC cell lines, the IFN-γ-induced PD-L1 mRNA and protein levels were significantly abrogated by *WDR5* or *MLL1* knockdown (not by *c-MYC* silencing), as well as by OICR-9429 (a highly selected and potent antagonist of WDR5 interactions with MLL1, c-MYC, and other partners) [14]. Some cell cycle, anti-apoptosis, DNA repair, and immune-related genes (such as *AURKA*, *CCNB1*, *E2F1*, *PLK1*, *BIRC5*, *XRCC2,* and *CD274*) were directly regulated by WDR5 and OICR-9429 in a H3K4me3 (histone H3 Lysine 4 tri-methylation)- and *c-MYC*-dependent manner; *WDR5* knockdown and OICR-9429 could reduce *c-MYC* recruitment and cell proliferation, increasing apoptosis and chemosensitivity to cisplatin in vitro and in vivo [14].

EZH2 (enhancer of zeste homolog 2) is the methyltransferase catalytic subunit of the polycomb repressive complex 2 (PRC2), which trimethylates Lysine 27 of histone H3 (H3K27me3) to promote transcriptional repression [158]. In PC models [158], EZH2 inhibition activates a double-stranded RNA–STING (stimulator of interferon genes)–ISGs (interferon-stimulated genes) stress response in tumor cells, upregulating genes involved in antigen presentation, Th1 chemokine signaling, and interferon response, including PD-L1 (dependent on STING activation) [158]. Moreover, EZH2 inhibition substantially increased the intratumoral trafficking of activated CD8+ and CD4+ T cells (decreasing the relative number of regulatory T cells, Tregs) and increased M1 tumor-associated macrophages (TAMs) (decreasing the tumor-promoting M2 macrophages). This pathway reversed the resistance to anti-PD-1 therapy in B6-MYC-CaP PCs in vivo [158]. 

mRNA translation is modulated by the rate of the elongation phase of protein synthesis through the phosphorylation of eukaryotic elongation factor 2 (eEF2) by its Ca^2+^-dependent protein kinase (eEF2K), which promotes PD-L1 expression [159].

miRNAs can regulate PD-L1 at a post-transcriptional level. In cell lines, Chen et al. [26] found that miR-15a negatively regulated PD-L1 expression. Moreover, miR15a overexpression promoted the cytotoxicity of CD8+ T cells against PC cells via directly targeting PD-L1, also decreasing tumor cell viability, migration, and invasion. miR-15a mimics downregulated pathways involved in the epithelial–mesenchymal transition (EMT) and *RAS/ERK* signaling. In contrast, PD-L1 overexpression in miR-15a mimics-transfected PC cells reversed these phenotypes; PD-L1 may influence the tumor-suppressive activity of miR-15a and stimulate multiple malignant phenotypes by activating the *RAS/ERK* signaling [26]. The long non-coding RNA gene KCNQ1 overlapping transcript 1 (lncRNA KCNQ1OT1) sponged miR-15a to upregulate the expression of PD-L1, thus inhibiting the cytotoxicity of CD8+ T cells and promoting tumor evasion [26]. Knocking down KCNQ1OT1 lowered PD-L1 expression and inhibited the viability, migration, invasion, and EMT of tumor cells, favoring their apoptosis; moreover, it enhanced the cytotoxicity and proliferation of CD8+ T-cells, reducing their apoptosis.

Functional experiments have demonstrated that miR-195 and miR-16 influenced the PD-L1-associated apoptosis in a co-culture model of human PC cell lines and human T cells [73]. miR-195 and miR-16 enhanced the radiotherapy efficacy in PC cell lines by activating the cytotoxic T cell response (repressing T cell dysfunction), inhibiting myeloid-derived suppressor cells (MDSCs) and Tregs, and increasing the secretion of pro-inflammatory cytokines (such as IFN-γ, TNF-α, and IL-2) in the tumor microenvironment through a PD-L1-dependent pathway [73]. 

All the above-mentioned observations referred to acinar PCs. Epigenetic methylation could regulate the *JAK/STAT* pathway—which is involved in PD-L1 expression—also in non-acinar PC histotypes. Indeed, the abstract of Sun et al. reported that PD-L1 expression was increased in small cell neuroendocrine PCs (in human cases, PC cell line, and mice model under IFN-γ stimulation); the block of the *JAK1/STAT1* pathway inhibited PD-L1 expression, while demethylation suppressed *JAK1* signaling [115]. 

### 2.3. Epigenetic Regulation of PD-L1 Expression: Studies on Human Patients, Including Data from The Cancer Genome Atlas (TCGA) Database

The epigenetic control of PD-L1 expression has also been confirmed in some studies on human PC tissues, sometimes using data derived from the TCGA database. In these studies, PD-L1 expression was evaluated on tumor tissue by real-time polymerase chain reaction (RT-PCR) analysis [14,26,60,73,90,93] and/or by immunohistochemistry [14,46,83,90,93,116] (Table 3).

Some authors found that normal prostatic tissue showed lower levels of PD-L1 RNA [26] and PD-L1 promoter methylation (mPD-L1) (compared to PC samples) [90]. 

In the study of Xiong et al. [60], PD-L1 RNA levels were higher in PC metastases than in primary tumor specimens (*n* = 35), correlating with MLL3 either in their series or in cases derived from the TCGA database (*p* < 0.01) [60]. MLL3 and PD-L1 RNA levels positively correlated to PSA levels (not Grade Group, age, or stage) [60]. 

Unlike PD-L1 negative regulators (HDAC1, HDAC2, and HDAC3), the expression ranks of PD-L1 and its positive regulators (*EP300*, *CREBBP*, *IRF-1*, and *BRD4*) negatively correlated to Grade Group in another study, suggesting an increase of function during cancer progression [116]. PD-L1, *EP300*, and *CREBBP* were negatively associated with overall survival. PD-L1 and PD-1 expression inversely correlated to tumor purity and increased tumor-infiltrating immune cells [116].

In a large series [90], high mPD-L1 (*p* = 0.008) and high PD-L1 protein expression (pePD-L1) (*p* = 0.002) (analyzed as continuous variables) both correlated to shorter biochemical recurrence-free survival (BRFS) in multivariate analysis (compared to pePD-L1^low^/mPD-L1^low^): these results were not confirmed in the validation cohort. Patients with pePD-L1^high^/mPD-L1^low^ or pePD-L1^low^/mPD-L1^high^ showed intermediate BRFS. PD-L1 DNA methylation was associated with the pT stage (*p* < 0.001) and the Grade Group (*p* = 0.001). 

Another study [93] found that high PD-L1 expression and aberrant *CXCL12* methylation (mCXCL12) correlated to significantly shorter BRFS than either PD-L1^low^/mCXCL12^normal^ or PD-L1^high^/mCXCL12^normal^ cases. The aberrant mCXCL12 group included either hypo- or hyper-methylated cases, which were combined in the analysis. Concordant results were found between radical prostatectomies and biopsies; unlike the mCXCL12 profile, CXCL12 immunohistochemical expression was not associated with the outcome [93].

The novel lncAMPC transcript produced by the *RNF165* (RING finger protein 165) gene apparently promoted metastatic behavior and immunosuppression in PCs via LIF/LIFR stimulation. In mouse PC models, PD-L1 immunohistochemical staining positively correlated to the lncAMPC-activated LIF levels, while LIF inhibition weakened the PD-L1-mediated immunosuppression in PC. The TCGA dataset analysis on human patients confirmed that PD-L1 expression was positively associated with lncAMPC-activated LIF levels and *RNF165* gene transcripts [108]. 

The epigenetic control of miRNAs concerning PD-L1 expression has also been investigated in some studies on PC patients. In the series of Tao et al. [73] (*n* = 40), miR-195 and miR-16 expression inversely correlated to PD-L1, PD-1, CD80, and CTLA-4 levels, showing a potentially positive association with longer BRFS [73]. In silico analysis of GSE21032 dataset (*n* = 131) confirmed that high miR-195/miR-16 levels were negatively associated with PD-L1 expression: both miRNAs also correlated to longer BRFS [73]. An inverse correlation between miR-15a expression and PD-L1 mRNA has been observed in another cohort of 30 PC tissues [26]. 

In contrast, miR-197 and miR-200a-c positively correlated to PD-L1 mRNA levels, being inversely associated with the methylation of PD-L1 promoter in a large series [90], suggesting that mPD-L1 is correlated to decreased mRNA expression, destabilizing miR-197 and miR-200a-c. miR-570 was only associated with mPD-L1, while miR-34a inversely correlated to mPD-L1 and mRNA expression [90]. miR-513 was not differentially expressed with regard to methylation and PD-L1 mRNA expression [90].

While PD-L1 levels were usually evaluated by RT-PCR analysis, some authors found that miR-424-3p (analyzed by in situ hybridization) significantly correlated to CTLA-4 (*p* < 0.001) and PD-L1 (*p* = 0.040) immunohistochemical expression in tumor cells [46,83].

In another series (cohort 1 = 136 PCs + 26 adjacent prostate tissue; cohort 2 = 126 PCs + 19 adjacent prostate tissue), the positivity rate of PD-L1 by immunohistochemistry (clone E1L3N, Cell Signaling Technology) was significantly higher in samples showing *WDR5* overexpression: the TCGA database (*n* = 374) also demonstrated a positive correlation between WDR5 and PD-L1 mRNA levels [14]. 

## 3. Discussion

Epigenetic changes may drive drug resistance. Regulation of epigenetic mediators of acquired tumor immune escape (such as EZH2, DNMTs, HDACs, Bromodomain and ExtraTerminal (BET) family members, and Lysine-specific demethylases) may overcome the immunotherapy resistance in PCs [63,77,158,159,160,161]. Indeed, PD-L1 expression is controlled at multiple levels, including genetic aberrations and epigenetic, transcriptional, or post-transcriptional regulations (such as DNA methylation, histone modifications, changes in gene transcription, mRNA stability, miRNAs, etc.) [159].

DNA methylation (mDNA) is involved in cell differentiation and is often aberrantly deregulated in cancers, favoring PC tumorigenesis and progression [5,14,78,90,97]. mDNA analysis is a promising diagnostic tool for specimens with a limited DNA quantity and/or in the case of formalin-fixed paraffin-embedded tissue samples with degraded DNA [90,97]. However, routine tests cannot identify the heterogeneous methylation patterns of the different cell types of a specimen; instead, microdissection techniques may be helpful [90,97].

DNA promoter methylation is frequently correlated to gene silencing [5,14]. In normal tissues, about 80% of CpGs DNA sequences are methylated, while CpG islands in the promoter regions of active genes are hypomethylated. Cancers (such as PC) usually shift the mDNA pattern toward a global hypomethylation, but CpGs in promoter regions of tumor suppressor genes may undergo hypermethylation, resulting in the inhibition of gene expression and gene loss of function. Complex aberrant methylation may also occur. Moreover, genes frequently silenced in the normal human genome (such as “long interspersed nuclear element-1”, *LINE-1*) can be re-expressed in PC cells [6,7,8,9,10,11,90,97,162,163,164]. 

The DNMTs family comprises DNA-modifying enzymes epigenetically regulating gene expression: they catalyze the addition of a methyl group to 5-methylcytosine (5mC) in CpG-enriched islands of gene-promoter regions (where they bind with a specific zinc finger domain) [6,7,8,9,10,11,162,163,164]. Cytosine methylation in mammals seems established by the complex recruitment of at least three independently encoded DNMTs (DNMT1, DNMT3a, and DNMT3b) [7,164]. DNMT1 keeps the mDNA pattern during replication, while DNMT3a/3b are de novo methyltransferases. Ten–eleven translocation enzymes can reverse mDNA. The three DNMTs coordinate mRNA expression in normal tissues and overexpression in cancers of various sites (colon, prostate, breast, liver, leukemia, etc.), typically promoting gene silencing. Decreased DNMT1 levels appear to be protective [165,166,167]. In PCs, DNMTs (especially DNMT3a and DNMT3b) were highly expressed, being associated with tumor progression [164]. An assessment of mDNA-based panels along with PSA screening demonstrated a highly predictive value for recurrence detection in PC patients [164]. 

In some studies, mPD-L1 suppressed PD-L1 expression in PCs [7,90,93]. In a large series, despite some limits, high mPD-L1 seemed an independent prognostic biomarker for BRFS in PC patients after radical prostatectomy, being also associated with the pT stage and Grade Group [90]. DNMT1 and DNMT3b may cooperatively maintain mDNA and gene silencing in cancer cells, synergizing their biological function, improving methylation efficacy, and suppressing PD-L1 expression more efficiently than DNMT3ac alone; this hypothesis was supported by pre-clinical studies using recombinant constructs (expressing the C-terminal domains of DNMT3a and/or DNMT1 fused with a zinc finger domain specifically binding to the PD-L1 promoter) [7].

The C-X-C chemokine receptor type 4 (CXCR4) and its endogenous ligand CXCL12 are expressed in various tumors [168]. They seemed to be involved in favoring androgen-dependent proliferation, tumor cell motility, and metastatic growth in PC [169], co-operating with the PD-1/PD-L1 pathway to suppress anti-cancer immunity [170]. CXCR4 expression favors chemotactic cell migration toward compartments releasing high levels of CXCL12, such as bone marrow (a frequent PC-metastatic site) [171]. Constant CXCL12 production causes CXCR4a downregulation and desensitization, resulting in a resting state of tumor cells and antagonizing the metastatic process [171]. CXCL12 promoter hypermethylation downregulates CXCL12 protein expression in PC, disrupting the cellular feedback internalization of membranous CXCR4 and so favoring tumor cell motility and metastatic potential [93]. In the series of Goltz et al. [93], high PD-L1 expression and aberrant mCXCL12 were associated with significantly shorter BRFS than either PD-L1^low^/mCXCL12^normal^ or PD-L1^high^/mCXCL12^normal^ cases. 

Epigenetic methylation may regulate the *JAK/STAT* pathway (involved in PD-L1 expression) also in non-acinar PCs (such as neuroendocrine carcinomas); however, limited data are available [115]. 

Different cancer types show aberrant expressions of HDACs, representing a promising target for cancer therapy [172]. Androgen receptor (AR) is a driver of PC progression: its downstream signaling events are closely regulated by epigenetic modifications [116]. HDAC inhibition could downregulate AR protein levels and significantly induce PD-L1 expression by increasing the acetylation of the *CD274* promoter, resulting in an immune-evasive microenvironment for tumor progression [173]. 

p300 is a coactivator of AR, regulating its transcriptional program and signaling axis and being involved in PC recurrence and chemoresistance. p300 directly acetylates AR or binds to AR, enhancing its transcriptional activity, inducing the expression of oncogenes, and promoting tumor growth [174]. Moreover, p300 could prevent AR protein degradation [174]. p300 is also involved in PC progression through PD-L1 upregulation, favoring tumor immune escape. The transcription factor IRF-1 could recruit p300 to the *CD274* promoter, inducing its transcription via histone acetylation. In an experimental study [116], the p300 inhibitor but not the anti-PD-L1 antibody significantly enhanced the efficacy of HDAC inhibitors on limiting tumor progression by blocking the HDAC inhibition-induced PD-L1 expression. Data on human patients and the TCGA dataset suggested that PD-L1 and p300 expression (unlike HDACs) negatively correlated to the Grade Group and overall survival, favoring an increase of function during cancer progression [116].

A485 (p300/CBP catalytic inhibitor) can decrease the proliferation of hematological tumors and AR-positive PCs [175]. A485 may enhance the efficacy of anti-PD-L1 antibody treatment, reducing the PD-L1 expression and exosomal secretion by PC cell lines; combined treatments inhibited the androgen-independent metastatic tumor growth in syngeneic PC models [116].

HDAC inhibitors have been approved for the treatment of T-cell lymphoma (vorinostat, SAHA; belinostat; and romidepsin) or multiple myeloma (panobinostat and LBH589). They have also been proposed in clinical trials for the treatment of solid malignancies (including PC) despite poor clinical responses in some cases [173,176].

Transcription factors usually bind to distal cis-regulatory regions (enhancers), regulating gene expression in normal conditions and during cancer development or progression [60]. MLL3 and MLL4 are huge molecular weight mono-methyltransferases of the MLL/COMPASS family; their regulation is still largely unknown. MLL3 and MLL4 favor the activity of enhancer regions (such as that of PD-L1) by the methylation of histone H3 on Lysine 4 and through the recruitment of other coactivators (such as p300). PD-L1 and MLL3 seemed positively correlated in PC patients and pre-clinical models [60]. 

The histone methylation regulator WDR5 is an important component of the SET1/MLL histone-methyltransferase complex and a critical co-activator of oncogenic pathways via the H3K4me3/*c-MYC*-dependent transcriptional activation of target genes, favoring tumor proliferation, metastases, chemoresistance, and AR-mediated castration-resistance [14]. WDR5 activates cell cycle, DNA repair, anti-apoptosis, and PD-L1 signaling, promoting PC progression; WDR5 seems an independent prognostic factor for progression-free survival and overall survival in PC. In PC cells, the IFN-γ-induced PD-L1 expression is blocked by *WDR5* or *MLL1* knockdown (not by *c-MYC* silencing) or by OICR-9429, suppressing proliferation and enhancing apoptosis, sensitivity to cisplatin, and immunotherapy [14].

EZH2 is overexpressed in PCs, contributing to tumor initiation and progression, and negatively regulating interferon-stimulated genes, including Th1-type chemokines, immune checkpoint molecules, and the major histocompatibility complex (MHC) [158]. Increased EZH2 function may favor immunosuppressive tumor microenvironments and immunotherapy resistance [158]. In PC models, EZH2 inhibition upregulated PD-L1 (dependent on STING activation) and other genes involved in antigen presentation, Th1 chemokine signaling, and interferon response. It also increased the intratumoral trafficking of activated CD8+ T cells and M1 TAMs, overall reversing the resistance to anti-PD-1 inhibitors. EZH2 actually regulates CD4+ T and Tregs differentiation [158,177,178]. In Tregs, the loss of *EZH2* resulted in the degradation of FOXP3, allowing the reprogramming of Tregs to T-helper cells [179]. It did not change the total number of intratumoral Tregs, but significantly increased the intratumoral CD4+ and CD8+ T cells. EZH2 inhibition in CD8+ T cells may induce PD-1 downregulation and increase cytotoxic activity [179]. MDSCs secrete IL-23 and may activate AR signaling at least in a subset of castration-resistant PCs, representing an important component of the tumor immunosuppressive microenvironment [161]. The EZH2 inhibitor did not dramatically alter intratumoral MDSCs, while significantly reprogrammed TAMs infiltrates, decreasing tumor-promoting M2 TAMs and increasing tumor-inhibiting M1 TAMs [159,161,179].

mRNA translation is regulated by the rate of the elongation phase of protein synthesis through the eEF2K-dependent phosphorylation of eEF2. eEF2K is activated under stress conditions, while it is inhibited by the anabolic mechanistic target of the rapamycin complex 1 (mTORC1) signaling pathway. eEF2K plays a role in cancer cell survival (decreasing protein synthesis and energy/amino acids consumption during nutrient depletion) and migration, angiogenesis, and the synthesis of integrins and other proteins. eEF2K also promotes PD-L1 expression in PC [159].

Bromodomains are protein domains recognizing acetylated Lysine residues on histone tails and other nuclear proteins, promoting gene transcription. The BET protein family comprises four transcriptional coactivators of cell cycle, regulating apoptosis, migration, and invasion (BRD2, BRD3, BRD4 and the testis-specific isoform BRDT). They are frequently overexpressed in various tumors, enhancing the transcription of oncogenic drivers (such as *AR* and *ERG*) in PC. BRD4 directly associates with P-TEFb (positive transcription elongation factor b) or interacts with DNA-specific transcription factors (p53, c-MYC, AR, ERG, etc.) [180]. The inhibition of BRD4 reduces the levels of AR-driven target genes in PC, decreasing tumor burden in murine models. BRD4 also regulates immune networks, as it reduces PD-L1 expression by directly binding to the PD-L1 promoter (mediating its transcription). It also increases MHC class I expression and alters the expression of immune-related genes. Moreover, it increases the number of tumor-infiltrating lymphocytes and susceptibility to the CD8+ T cell-mediated cytotoxicity [123,127,180,181,182,183]. 

In some pre-clinical studies, treatment with JQ1 (bromodomain inhibitor) suppressed PD-L1 expression [123,127]. JQ1 stimulates the antigen presentation pathways promoted by IFN-γ. JQ1 upregulates TAF9, a subunit of the Transcription Factor IID (TFIID) required for the initiation of transcription by RNA Polymerase II. TAF9 associates with CIITA (MHC class II transactivator), forming a complex responsible for MHC class I gene upregulation after IFN-γ stimulation. JQ1 differentially modulates the expression of MHC class I alleles, and it may limit the transcription of inhibitors of RelA/NF-kB, thus enhancing their ability to bind selectively to HLA-A and B and increasing mRNA and protein levels. A combined treatment with bromodomain inhibitors and IFN-γ upregulated TRIM36 (E3 ubiquitin-protein ligase) in a dose-dependent manner. Increased TRIM36 expression was associated with the inhibition of PC proliferation and cell-cycle progression through the inhibition of the *MAPK/ERK* pathway [123,184]. TRIM36 is also involved in antigen processing [32]. 

Preclinical models documented resistance to BET inhibitors through largely unknown molecular mechanisms. Speckle-type POZ protein (SPOP) is a E3 ubiquitin ligase of the MATH–BTB family, containing MATH and C-terminal BTB domains, which are both required for BRD4 ubiquitination and degradation. In PC, *SPOP* or *BRD4* mutations confer resistance to BET inhibitors; they inhibit the SPOP-mediated BRD4 destruction by disrupting the SPOP–BRD4 interaction, stabilizing BRD4, and leading to its cooperation with AR, ERG, and other oncogenic transcription factors [180]. 

miRNAs are small non-coding RNAs regulating gene expression and the metabolic/signaling pathways involved in cell proliferation, differentiation, and survival. They cause mRNA translational inhibition and/or degradation at a post-transcriptional level. miRNAs are potential biomarkers for PC metastasis, apoptotic resistance, and AR signaling disruption [12]. However, miRNA levels vary across different tissues and cancer specimens; heterogeneous expression can be also found in the same tumor [180]. 

miRNAs regulate PD-L1 expression, modifying the downstream processing of PD-L1 mRNA. Few studies on cell lines and human PC tissues suggested a potential correlation between PD-L1 and some miRNAs (miR-195, miR-15, miR-16, miR-197, miR-200, miR-570, miR-34a, and miR-424) in PC. However, further studies are required.

miR-195, miR-15 and miR-16 are members of the miR-15/-16/-195/-424/-497/-503 family; preclinical models and rare studies on human patients suggested that they downregulate PD-L1 expression in PC. miR-195 inhibits PC progression by targeting the *RPS6KB1* gene (encoding the ribosomal protein S6 serine/threonine kinase B1), which is involved in mTOR signaling, promoting protein synthesis, cell growth, and cell proliferation. miR-15a and miR-16 act as tumor suppressors: they downregulate multiple oncogenes (*BCL2*, *MCL1*, *CCND1*, and *WNT3A*), decreasing PC cell survival, proliferation, invasion, and EMT by targeting TGF-β signaling. These miRNAs also regulate angiogenesis, metastatic potential, chemoresistance, and the tumor microenvironment crosstalk in PC [12,26,69,73,185,186,187,188,189,190,191,192,193]. 

In a study, miR-195 and miR-16 expression were inversely associated with PD-L1, PD-1, CD80, and CTLA-4 levels, potentially favoring a longer BRFS. They may regulate cytokine secretion in the tumor microenvironment through a PD-L1-dependent pathway and influence the PD-L1-associated apoptosis in PC cell lines [69]. In addition, miR-195 and miR-16 overexpression increases the radiosensitivity of cancer cells; in vitro and in vivo restoration of their expression enhanced radiotherapy via T cell activation in the tumor microenvironment by blocking the PD-L1 immune checkpoint in PC cells, suggesting a synergistic effect of immunotherapy and radiotherapy [73]. 

LncRNA transcripts may represent useful prognosticators of PC metastasis and proliferation [12]. LncAMPC (a *RNF165* transcript) seemed to promote metastasis and immunosuppression in PC by stimulating LIF/LIFR expression: in TCGA datasets, PD-L1 expression positively correlated to the lncAMPC-activated LIF level and *RNF165* gene transcripts [104]. Among various targets, miR-15a may bind to the 3′-UTR of PD-L1 and to the lncRNA KCNQ1OT1, located into the same sequence of miR-15a and involved in promoting oncogenic phenotypes and chemoresistance in multiple cancers (colon, lung, breast, liver, etc.). In PC cell lines, KCNQ1OT1 sponged miR-15a, suppressing the miR-15a-mediated inhibition of PD-L1, thus leading to PD-L1 upregulation, the inhibition of CD8+ T-cells cytotoxicity, and the promotion of tumor evasion [26]. The mechanism responsible for KCNQ1OT1 upregulation is still unknown, while the KCNQ1OT1/miR-15a/PD-L1 axis apparently promotes *RAS/ERK* signaling activation, inducing tumor immune evasion. *ERK* signaling may directly activate PD-L1 transcription, stabilizing PD-L1 mRNA as in other contexts [26,194,195,196].

miR-197 and miR-200a-c positively correlated to PD-L1 mRNA levels and were inversely associated with mPD-L1 in a large PC series, suggesting that mPD-L1 was associated with lower mRNA levels destabilizing miR-197 and miR-200a-c [90]. miR-197 expression was rarely investigated in PC: it seems involved in the proliferation, invasion, and metastatic potential of PC cells by regulating integrin subunit alpha V (ITGAV) expression through the STAT5 pathway [197]. miR-197–3p is apparently involved in *PI3K/AKT3* signaling, possibly favoring castration-resistance by targeting *RAS*, *RHO*, and the SCF complex [198]. It also represses PC cell proliferation by regulating the voltage-dependent anion channel 1 (VDAC1)/AKT/β-catenin axis [199]. Circular RNA itchy E3 ubiquitin-protein ligase upregulation may suppress cell proliferation, promoting apoptosis by targeting miR-197 in PC [200]. Moreover, miR-197 regulates AR protein levels and activity in PC cells [200]. In a PC study, miR-197, miR-346, and miR-361-3p downregulated two AR corepressors (*ARHGDIA* and *TAGLN2*) and upregulated the *YWHAZ* oncogene [201].

The miR-200 family inhibits EMT, cancer growth, invasion, and metastasis via the inhibition of ZEB1 and ZEB2 (transcriptional regulators of E-Cadherin). The loss of the miR-200 family through mDNA results in aggressive PC features. PDGF (involved in EMT) and other growth factors regulate miR-200 expression, while miR-200c directly influences the EGFR and TGF-β receptor signaling [202,203,204]. miR-200b seems significantly downregulated in castration-resistant PCs: miR-200 influences Notch1 expression, and together they regulate EMT progression [205]. miR-200c expression is negatively regulated by ERG in PC cells lines [206]. miR-200 overexpression can reduce PC growth and modulate chemosensitivity [207].

In a PC sudy, miR-570 was only associated with mPD-L1, while miR-34a was inversely correlated to mPD-L1 and mRNA expression. miR-513 was not differentially expressed with regard to methylation and PD-L1 mRNA expression [90]. miR-34a, miR-143, miR-148a, and the miR-200 family are involved in chemoresistance by the inhibition of apoptosis and the activation of signaling pathways. miR-34a expression levels were decreased in androgen-resistant PC3 and DU145 cell lines (vs. androgen-sensitive LNPCa and normal prostatic tissue). *TP53* involvement in miR-34a/miR-34c-mediated apoptosis was found in AR+ PC cells: miR-34a expression appeared completely absent in p53-null PC3 cells [208,209].

In a study, miR-424 was highly expressed in metastatic subclones of DU145 cell lines, while the effects on EMT promotion were controversial [210,211]. The PD-1/PD-L1 pathway regulates T cell activation during inflammatory processes, while CTLA-4 is a protein receptor on T cell surface, inhibiting T cell activity during the priming phase; miR-424 may inhibit the PD1/PD-L1 and CD80/CTLA-4 activity, inducing tumor suppression [46]. In PC patients, miR-424–3p expression by in situ hybridization significantly correlated to CTLA-4 (*p* < 0.001) and PD-L1 (*p* = 0.040) immunohistochemical positivity of tumor cells. Low miR-424–3p expression was significantly correlated with reduced clinical failure-free survival, aggressive PC features (high Grade Group, large tumor size, perineural and vascular invasion), and CTLA-4/PD-L1 expression on tumor cells, while no association with T cells subsets was found. CTLA-4 was associated with CD3+ and CD4+ T cells and PD-1 expression by tumor cells and stroma (considered separately or as one compartment) [46,83].

Recently, some authors reported that patients with advanced PC release fully functional circulating extracellular vesicles containing miR-424, which facilitate the acquisition of stem-like traits by low tumorigenic cells, favoring metastatic behavior and cancer progression; circulating miR-424-expressing extracellular vesicles were more frequent in patients with metastatic PCs [212,213,214].

## 4. Materials and Methods 

Systematic literature reviews (SLRs) and meta-analyses have become increasingly important in health care as: (1) clinicians read SLRs to keep themselves up to date; (2) they are often a starting point for the development of clinical guidelines or further studies/trials; (3) granting agencies may require the results of SLRs to ensure the justification for research financial support. For these reasons, impacted healthcare journals frequently ask contributing authors to conduct their SLRs according to the PRISMA guidelines (http://www.prisma-statement.org/; accessed on 8 May 2021), which include an evidence-based minimum set of items for reporting and are useful for a critical evaluation of the submitted manuscripts. So, we have conducted our SLR according to these guidelines, searching in multiple databases, as previously described in the various topics/contexts in which they are applicable [215,216,217,218,219,220,221,222,223,224,225,226,227,228,229,230,231,232,233,234,235,236,237,238,239,240,241,242,243,244,245,246,247,248,249]. 

Our study aimed to answer the following PICO (population, intervention, comparison, outcomes) questions:

- Population: patients, tumor cell lines, or mouse models included in studies concerning the role of PD-L1 in PC.
- Intervention: any type of treatment.
- Comparison: no comparisons are expected.
- Outcomes: patient’s status at last follow-up (no evidence of disease, alive with disease, or dead of disease), response to therapy, biochemical recurrence-free survival, metastasis-free survival, cancer-specific survival, disease-free survival, clinical failure-free survival, overall survival, and progression-free survival. As regards experiments on PC cell lines and mouse models: any reported effect on cancer and immune cell migration, proliferation, viability, growth, resistance/response to therapy, cytotoxic/anti-tumor activity, PD-L1 expression, and mice/cell line survival.

Study design: retrospective observational study (case series/reports, clinical trials, and experimental studies). 

Eligibility/inclusion criteria: experimental studies (tumor cell lines and mouse models) or clinic-pathologic studies on human patients concerning the role PD-L1 in PCs.

Exclusion criteria: tumors not arising from the prostate; non-carcinomatous histotypes; studies not examining PD-L1; cases with uncertain diagnosis; and review articles without new cases.

Information sources and search strategy: we searched for “PD-L1 AND (prostate OR prostatic) AND (adenocarcinoma OR adenocarcinomas OR cancer)” in PubMed (all fields), Web of Science (Topic/Title), and Scopus (Title/Abstract/Keywords) databases. No limitations or additional filters were set. The bibliographic research ended on 8 May 2021.

Study selection: two independent reviewers selected the studies using a 2-step screening method. In the first step, the screening of titles and abstracts was performed to verify the eligibility/inclusion criteria and exclude irrelevant studies. In the second step, full texts of the selected articles were screened by 2 reviewers to verify the eligibility/inclusion criteria and to avoid duplication of the articles. Two other authors screened the reference lists to look for additional relevant publications. Finally, two authors checked the extracted data. 

Objects of the systematic review: (1) to update and summarize the literature concerning the role of PD-L1 in PC cells, and (2) to report any information regarding clinic-pathological features, treatment strategies, and patients’ outcomes. 

Data collection process/data items: data collection was study-related (authors and year of study publication) and case-related (tumor stage at presentation, Grade Group, type of specimen, treatment, test methods and results of PD-L1 expression, follow-up and outcomes, and experiment type). 

Statistical analysis: the collected data were reported as continuous or categorical variables. Categorical variables were summarized by frequencies and percentages; continuous variables were summarized by ranges, mean, and median values. Time-to-recurrence was the time from the primary treatment to the disease recurrence. The survival status was the time from the primary treatment to the last follow-up. 

To better present the results of our SLR and discuss the multiple interesting facets of PD-L1 expression by PC in detail, we have divided the presentation and discussion of our results into different articles, representing independent, self-sufficient chapters/parts of our work. They highlight various subtopics, including PD-L1 immunohistochemical expression in PC with a discussion of pre-analytical and interpretation variables; the clinic-pathological correlations of PD-L1 expression in PC; PD-L1 intracellular signaling pathways in PC and the influence of the tumor microenvironment; the data of pre-clinical studies (cell lines and mouse models) about the effects of experimental treatments on PD-L1 expression by PC cells; an investigation of the correlations of PD-L1 expression with the status of the mismatch repair system, *BRCA*, *PTEN,* and other main genes in PC; PD-L1 expression in liquid biopsy samples; the information of clinical trials, etc. [250,251,252,253]. We direct the readers to these papers for further details. 

## 5. Conclusions 

Epigenetic alterations influence oncogene/oncosuppressor gene expression without changing the DNA sequence. PC displays a complex genetic and epigenetic regulation of cell growth pathways and tumor progression. 

In PC cell lines, CpG island methylation of the *CD274* promoter negatively regulated PD-L1 expression. Histone modifiers are involved in the PD-L1 transcription rate: the deletion or silencing of histone modifiers (such as MLL3 and MML1) may regulate PD-L1 expression. 

Epigenetic drugs could be promising in reprogramming tumor cells, reversing epigenetic modifications, and cancer immune evasion. Drugs promoting a chromatin-inactive transcriptional state (such as bromodomain or p300/CBP inhibitors) reduced PD-L1 expression, while those favoring a chromatin-active state (i.e., histone deacetylase inhibitors) increased PD-L1 expression. 

miRNAs can regulate PD-L1 at a post-transcriptional level. miR-195/miR-16 were negatively associated with PD-L1 expression and positively correlated to longer BRFS. They also enhanced the radiotherapy efficacy in PC cell lines. miR-197 and miR-200a-c were positively correlated to PD-L1 mRNA levels and were inversely associated with the methylation of PD-L1 promoters in a large series. miR-570, miR-34a and miR-513 may also be involved in the epigenetic regulation of PC.

## Figures and Tables

**Table 1:** PD-L1 epigenetic regulation in prostatic carcinoma-derived cell lines.

		Experiment Type	Cell Lines	Effects on PD-L1 Expression	Possible Mechanism of Action
DNMT	Dnmt1, Dnmt3 [7]	Methyltransferase overexpression	DU145	Neg	NR
Histone modifiers	*MLL3* [60]	*MLL3* deletion	PC3, TRAMP-C2	Pos	NR
	*MML1* [14]	*MLL1* silencing	PC3, DU145	Pos	NR
	HDAC class I [116]	HDAC class I deletion	DU145	Neg	NR
	p300/CBP [116]	*EP300* or *CBP* deletion	DU145	Pos	NR
miRNAs	miR-195, miR-16 [73]	miRNA overexpression	PC3, DU145, TRAMP-C1	Neg	NR
	miR-15 [26]	Co with TTC with miRNA mimic or inhibitor	PC3, DU145 (*)	Neg	Reduction of cell viability, migration and invasion, and increased apoptosis of tumor cells. Increased CD8+ T cell cytotoxicity when miR-15 is overexpressed. Opposite effects when miR-15 is inhibited.
Long non-coding RNA	KCNQ1OT1 [26]	Co with long non-coding RNA overexpressed TTC	PC3, DU145 (*)	Pos	Increased cell viability, migration, invasion and apoptosis of tumor cells, reduction of CD8+ T cell cytotoxicity.
mRNA translation modulators	eEF2K [159]	eEF2K ablation	PC3	Pos	eEF2K promotes the association of PD-L1 mRNAs with translationally active polyribosomes, enhancing PD-L1 expression. eEF2K-depleted cancer cells are more vulnerable to NK cells.

(*): effects on CD8+ T-cell infiltrate were also investigated. Co: Co-culture; DNMT: DNA methyltransferase; HDAC: Histone deacetylase; Neg: Negative regulator; NR: not reported (no effect was investigated); Pos: Positive regulator; TTC: transfected tumor cells.

**Table 2:** Experimental treatments with epigenetic drugs.

Drug	Drug Type	Experiment Type	Cell Lines	Effects on PD-L1	Studied Effect
JQ1 [127]	bromodomain inhibitor	Treatment	PC3	↓	↓ Proliferation
JQ1 [123]	bromodomain inhibitor	Treatment	PC3, DU145, Myc-Cap	↓	NEI
RVX [123]	bromodomain inhibitor	Treatment	PC3	↓	NEI
SAHA [116]	HDAC class I-II inhibitor	Treatment	PC3, DU145	↑	NEI
LBH589 [116]	pan-deacetylase inhibitor	Treatment	PC3, DU145	↑	NEI
A485 [116]	p300/CBP inhibitor	Treatment	TRAMP-C2 Ras	↓	NEI
OIRC-9429 [14]	WDR5 inhibitor	Treatment	PC3, DU145	↓	NEI
EZH2 inhibitor [158]	EZH2 inhibitor	Treatment	MYC-CaP	↑	↑ genes involved in antigen presentation, Th1 chemokine signaling and IFN response ↑ CD8+ and CD4+ T cells ↓ Tregs ↑ M1 TAMs ↓ M2 TAMs

↓ Downregulation or decrease; ↑ Upregulation or increase; IFN: interferon; NEI: No effect was investigated; TAMs: tumor-associated macrophages; Tregs: regulatory T cells.

**Table 3:** Epigenetic regulation of PD-L1: human studies on prostatic adenocarcinoma.

Ref.	Samples	GG/Stage	Results
[26]	30 PC 30 BPT	NR	Inverse correlation between PD-L1 and miR-15a levels. Significantly higher KCNQ1OT1, PD-L1, and CD8 expression in PCs than BPT (lower miR-15a levels).
[60]	66 mCRPC (RP, MTS)	GG: 1–5 Stage: I–IV	MLL3 and PD-L1 RNA levels positively correlated to PSA (not GG, age or stage). In 23 paired castration-resistant PC samples, higher MLL3 and PD-L1 RNA levels in MTS than in primary PC samples.
[73]	40 PC (ff) 20 BPT (ff)	NR	miR-195 and miR-16 expression inversely correlated to PD-L1 levels in PC. High miR-195/miR-16 levels correlated to longer BRFS.
[90,93]	TC: 498 PC (TCGA); VC: 299 PC (RP)	GG: 1–5 Stage: pT2–4 Nx/0/1	Lower levels of mPD-L1 in normal prostate (vs. PC). On multivariate analysis, mPD-L1^high^ (HR = 1.22 [95%CI: 1.05–1.42] *p* = 0.008) and pePD-L1^high^ (HR = 2.58, 95% CI: 1.43–4.63; *p* = 0.002) (analyzed as continuous variables) correlated to shorter BRFS (vs. pePD-L1^low^/mPD-L1^low^) in TC (not in VC). pePD-L1^high^/mPD-L1^low^ or pePD-L1^low^/mPD-L1^high^ showed intermediate BRFS. mPD-L1 corelated to pT stage (*p* = 0.010) and GG (*p* = 0.001). miR-197 and miR-200a-c positively correlated to PD-L1 mRNA levels and inversely correlated to mPD-L1 (possible association of mPD-L1 with decreased mRNA levels destabilizing miR-197 and miR-200a-c). miR-570 correlated to mPD-L1. miR-34a inversely correlated to mPD-L1 and mRNA expression. Trend toward an association of PD-L1 with mCXCL12 (Spearman’s rank correlation; ρ = 0.132, *p* = 0.084). Kaplan–Meier analysis: PD-L1^low^ PCs (*n* = 85) showed the longest BRFS (mean 112 months), PD-L1^high^/mCXCL12^medium^ PCs (*n* = 45) had the best BRFS (mean 107 months), PD-L1^high^/mCXCL12^low^ (*n* = 15) and PD-L1^high^/mCXCL12^high^ (*n* = 27) PCs showed short BRFS (mean 52 and 83 months, respectively; *n* = 151, χ^2^ = 12.99; *p* = 0.005).
[116]	495 (TCGA)	NR	*EP300* (p300), *CREBBP* (CBP), *KAT2B* (PCAF), and *BRD4* positively correlated to *CD274* expression (*p* < 0.001) (comparable with IRF-1). *CBP* showed stronger correlation with PD-L1 expression than p300. No correlation between HDAC2/3 with *CD274* (*p* > 0.05). *CD274*, *EP300*, *CREBBP*, *IRF1,* and *BRD4* ranks negatively correlated to GG, while negative regulators of PD-L1 expression (HDAC1, HDAC2 and HDAC3) seemed insignificant during the cancer progression. *CD274*, *EP300* and *CREBBP* negatively correlated to overall survival. Expression levels of *CD274* and *PDCD1* were associated with increased number of tumor-infiltrating immune cells.
[108]	NR (TCGA)	NR	In TCGA series, PD-L1 expression was positively associated to lncAMPC (long non-coding RNA NR_046357.1)-activated LIF levels and *RNF165* gene transcripts.
[46,83]	535 (RP) (°)	GG: 1–5 Stage: pT2–3b	Significant correlations between mir-424-3p (in situ hybridization) and the following: high GG (r = 0.12, *p* = 0.014); GG ≥8 (r = 0.11, *p* = 0.024); large tumor size (>20 mm, r = 0.13, *p* = 0.013); perineural infltration (r = 0.11, *p* = 0.030); vascular invasion (r = 0.12, *p* = 0.014); CTLA-4 (r = 0.10; *p* < 0.001); and PD-L1 (cut-off: mean value = 0; r = 0.11; *p* = 0.040) immunohistochemical expression by tumor cells (°). miR-424-3p did not correlate to any T cell subsets. CTLA-4 did not correlate to any clinicopathological variables, while it was associated with: PD-1 expression on tumor cells (r = 0,10, *p* = 0.054) and stromal cells (r = 0.16; *p* = 0.002); CD3+ (*p* = 0.028) and CD4+ (*p* = 0.009) T cells. On multivariate analysis, perineural infiltration, pT stage, pT3b, GG, GG ≥4, and positive surgical margins (either apical or non-apical) were significant for poor BRFS. Low miR-424-3p expression (HR: 0.44, 95% CI 0.22–0.87, *p* = 0.018) was associated with aggressive disease and poor clinical failure-free survival.
[14]	262 (RP) 374 (TCGA)	GG: 2-3 Stage: T2-4 N0-1	PD-L1 positivity rate (immunohistochemistry, clone E1L3N, Cell Signaling Technology) was significantly higher in samples showing WDR5 overexpression. The TCGA database confirmed a positive correlation between WDR5 and PD-L1 mRNA levels.

(°): for PD-L1 and CTLA-4 correlations, a previous series of 402 RP was used [83]. BPT: benign prostatic tissue; BRFS: biochemical recurrence-free survival; CI: Confidence Interval; ff: fresh frozen tissue; GG: Grade Group; HR: hazard radio; mCRPC: metastatic castration-resistant prostate cancer; mCXCL12: methylation of *CXCL12*; mPD-L1: methylation of PD-L1 promoter; MTS: metastases; NR: not reported; PC: prostate cancer; PCR: Polymerase chain reaction; pePD-L1: PD-L1 protein expression; Ref: reference; RP: radical prostatectomy; TC: training cohort; TCGA: The Cancer Genome Atlas database; VC: validation cohort.
